# Genetic- and fiber-diet-mediated changes in virulence factors in pig colon contents and feces and their driving factors

**DOI:** 10.3389/fvets.2024.1351962

**Published:** 2024-04-16

**Authors:** Tao Wang, Yuheng Luo, Xiangfeng Kong, Bing Yu, Ping Zheng, Zhiqing Huang, Xiangbing Mao, Jie Yu, Junqiu Luo, Hui Yan, Jun He

**Affiliations:** ^1^Institute of Animal Nutrition, Sichuan Agricultural University, Chengdu, China; ^2^Key Laboratory of Animal Disease-resistant Nutrition, Chengdu, China; ^3^Institute of Subtropical Agriculture, Chinese Academy of Sciences, Changsha, China

**Keywords:** virulence factors (VFs), pig, high fiber diet, colon, feces

## Abstract

Virulence factors (VFs) are key factors for microorganisms to establish defense mechanisms in the host and enhance their pathogenic potential. However, the spectrum of virulence factors in pig colon and feces, as well as the influence of dietary and genetic factors on them, remains unreported. In this study, we firstly revealed the diversity, abundance and distribution characteristics of VFs in the colonic contents of different breeds of pigs (Taoyuan, Xiangcun and Duroc pig) fed with different fiber levels by using a metagenomic analysis. The analysis resulted in the identification of 1,236 virulence factors, which could be grouped into 16 virulence features. Among these, Taoyuan pigs exhibited significantly higher levels of virulence factors compared to Duroc pigs. The high-fiber diet significantly reduced the abundance of certain virulence factor categories, including iron uptake systems (*FbpABC*, *HitABC*) and Ig protease categories in the colon, along with a noteworthy decrease in the relative abundance of plasmid categories in mobile genetic elements (MGEs). Further we examined VFs in feces using absolute quantification. The results showed that high-fiber diets reduce fecal excretion of VFs and that this effect is strongly influenced by MGEs and short-chain fatty acids (SCFAs). *In vitro* fermentation experiments confirmed that acetic acid (AA) led to a decrease in the relative abundance of VFs (*p* < 0.1). In conclusion, our findings reveal for the first time how fiber diet and genetic factors affect the distribution of VFs in pig colon contents and feces and their driving factors. This information provides valuable reference data to further improve food safety and animal health.

## Introduction

The gastrointestinal tract and fecal matter contain diverse microbial communities, with pathogenic microorganisms posing significant health risks to humans and animals. Approximately 61% of human pathogens can infect animals, emphasizing the global significance of zoonotic diseases (1). Pathogens enhance their defenses and pathogenicity within hosts using virulence factors (VFs) (2, 3), which play critical roles during infections and dictate the pathogens’ ability to cause disease. These VFs can directly interact with host tissues or help bacteria evade immune responses (4). Past research has elucidated mechanisms by which bacteria-driven VFs damage host cells, including disrupting cellular signaling, triggering nonspecific immune responses, and activating specific protease functions. For example, adhesins encoded by *Klebsiella pneumoniae*’*s FimA* gene promote microbial adhesion (5), whereas *Escherichia coli* produced toxins, such as hemolysins, can inhibit host cell functions or cause cell lysis (6). However, despite extensive clinical research on virulence factors carried by pathogenic microorganisms, studies on the distribution of VFs in the gastrointestinal tract and feces of food animals are still limited.

Livestock fecal matter is a direct product of gastrointestinal contents. Its physicochemical properties and microbial composition are influenced by dietary and gut factors (7). Altering dietary habits can significantly impact the microbial communities within the gastrointestinal tract and the VFs they possess, subsequently affecting VFs excretion in feces (8). For example, following infection with Citrobacter, a murine mucosal pathogen, in an experimental mouse model, dietary iron addition was found to induce a cooperative defense mechanism that weakens the virulence of the pathogen and promotes prolonged asymptomatic infections by triggering physiological changes in the host (9, 10). Given this, we hypothesize that changes in dietary fiber can alter the metabolic activities in the gastrointestinal contents, affecting the VFs present in gut microorganisms and influencing VF excretion patterns.

Historically, VFs research in pathogenic bacteria have largely depended on single-gene PCR or quantitative PCR (qPCR) methods (11, 12). However, these techniques face technical constraints, making a holistic and swift assessment of VF diversity in samples challenging. With advancements in microbial genomics, especially complete bacterial genome sequencing, metagenomics has become a crucial tool for identifying VFs. This approach aids in extensively studying VFs and understanding the evolutionary dynamics of VFs, considering various environmental selection pressures and gene gain or loss in specific pathogenic scenarios (13). In our study, we selected Taoyuan, Duroc and Xiangcun pig breeds as models and used a combination of high-through put metagenomics and qPCR to characterize the extent of VFs in the colon of different breeds of pigs at different fiber levels and to validate the excretion of VFs in the feces. And we used liquid chromatography and *in vitro* fermentation modeling to explore potential drivers of changes in VFs in colon contents and feces. The study aimed to characterize the dietary and genetically mediated distribution of VFs in porcine colonic contents and furthermore to research the drivers of fiber influencing the excretion of VFs in porcine feces. Our study provides important reference data for animal breeding and food health studies.

## Materials and methods

### Ethics statement

The management of animal experiments involved in the research shall refer to the “Regulations on the Administration of Laboratory Animals” (Ministry of Science and Technology, China, revised in June 2004). Sample collection was approved by the Institutional Animal Care and Use Committee of Sichuan Agricultural University, Sichuan, China (No. 20181105), and operated in strict accordance with ethical guidelines.

### Animal trial and sample collection

The experiment selected at 60 days of age Taoyuan pigs (Average weight: 13.87 ± 0.58 kg; Purchased from Xiangcun Hi-Tech Agriculture Co., Ltd.), Duroc pigs (Average weight 18.50 ± 1.09 kg; Purchased from Linli Tianxin Seed Industry Co., Ltd) and their cross-bred varieties Xiangcun pigs (Average weight 14.47 ± 0.15 kg; Purchased from Xiangcun Hi-Tech Agriculture Co., Ltd.), and adopted a 3 × 2 factorial design, that is, 3 varieties of pigs (20 pigs for each variety, that is, each 10 pigs in each treatment group, 60 pigs in total) were fed a high-fiber diet (crude fiber: 6–7%; digestible energy: 3.5%; crude protein: 19.16%.) and a low-fiber diet (crude fiber: 2–3%; Digestible Energy: 3.49%; Crude Protein: 19.15%). Wheat bran fiber was purchased from Chengdu Tubaite Technology Co., Ltd. (manufacturer: JRS, model: WF200, purity >95%). All pigs were housed in single pens, each pen was equipped with feeders and nipple drinkers, the room temperature was 28°C, water and food were free, and the experimental period was 28 days. Pigs were slaughtered on the last day of the experiment and colonic contents and feces were collected.

### Metagenome sequencing and data analysis

Paired-end libraries were constructed by NEXTFLEX Rapid DNA Seq (Bioo Scientific, Austin, TX, United States) using 1 μg of high-quality DNA constructs, and then sequenced using the Novaseq6000 platform from Shanghai Majorbio BioPharmaceuticals Biotechnology Co., Ltd. (Shanghai, China). Raw data were filtered using Trimmomatic v0.38 to remove reads containing more than three ambiguous nucleotides with an average quality score of <20 and to remove artificially duplicated reads. Clean reads were then assembled into contigs using MEGAHIT (14) (https://github.com/voutcn/megahit, version 1.1.2). Only overlapping clusters of ≥300 bp were retained for subsequent analysis. Open reading frames (ORFs) of all assembled overlap clusters were predicted using MetaGene and translated into amino acid sequences. A catalog of non-redundant genes was constructed using CD-HIT (15) (http://www.bioinformatics.org/cd-hit/, version 4.6.1) with 90% sequence identity and 90% coverage. High-quality reads were aligned to the non-redundant gene catalog to calculate gene abundance with 95% identity using SOAPaligner (http://soap.genomics.org.cn/, version 2.21). Virulence factor annotation was performed using Diamond (16) (http://www.diamondsearch.org/index.php, version 0.8.35 http://www.mgc.ac.cn/VFs/) *e* value cutoff was 1 × 10^−5^. Sequence data associated with this project have been deposited in the NCBI Short Read Archive database (Accession Number: PRJNA849732).

### SCFA determination

Weighed about 1 g of samples (colon contents and fecal samples) in a 2 mL centrifuge tube, added ultrapure water in the ratio of 1:2.5, mixed well and let it stand for 30 min. Took 2 mL of supernatant in a new centrifuge tube, and incubated for 30 min at 4°C and 12,000 r/min for 10 min. Took 1 mL of supernatant and added 0.2 mL of 25% (w/v) metaphosphoric acid solution, mixed well, and incubated for 30 min at 4°C for 10 min at 12,000 r/min. Added 0.2 mL of 25% (w/v) metaphosphoric acid solution to 1 mL of the supernatant, mixed well, and incubated at 4°C for 30 min. Incubated for 10 min at 12,000 r/min for 10 min. Took 0.6 mL of the supernatant and added 11.67 μL of crotonic acid solution (210 mmol/L) to the supernatant, mixed well, and then took 0.3 mL of supernatant into a new centrifugal tube, and then added 0.9 mL of methanol solution. The SCFA concentration in the samples was determined using a gas chromatography system (CP-3800 GC, Varian, Inc., Walnut Creek, CA, United States) and following the method of Franklin et al. (17).

### qPCR

DNA was extracted from feces and *in vitro* fermentation samples according to the instructions of the Fecal Genomic DNA Extraction Kit (D2700, Solarbio, China). The concentration and integrity of the DNA samples were determined by using a NanoDrop Microspectrophotometer (NanoDrop 2000, NanoDrop, United States) and Gel Imaging (GelDocXR, Bio-Rad, United States) to detect the concentration, purity and integrity of DNA samples. The quantitative analysis of VFs gene was performed by qPCR using fecal DNA as template (CFX96 real-time fluorescence quantification instrument, ABI7900, United States). Three replicates were set up for each sample, and the results were averaged. The reaction system consisted of 5 μL of SYBR Premix Ex Taq^™^ II, 0.5 μL of each of the upstream and downstream primers, 2 μL of the DNA template, and 2 μL of ddH_2_O. The abundance of 46 genes in the fecal samples was examined by qPCR (QS6FX, ABI, United States) (Supplementary Table S5) (11). Absolute abundance of VFs was calculated according to the method of Xie et al. (11).

### *In vitro* fermentation assay

*In vitro* fermentation experiments were conducted according to existing methods (18). Duroc pigs were in normal body condition for 1 month prior to fecal collection. The collected feces were strongly mixed under aseptic conditions with 32% (w/v) inoculum (fecal slurry) in phosphate buffer, pH adjusted to 7.0 with 0.1 M HCl, and then filtered through 4 layers of gauze. The filtrate was bubbled with N_2_. Then, 8 mL of fermentation medium, 2 mL of fecal inoculum and treatment agent were added to each 50 mL fermentation tube under anaerobic (N_2_) and aseptic conditions. The fermentation tubes were then incubated for 20 h at 37°C at 120 rpm. After 20 h of fermentation, the fermentation was stopped by immersion in ice water for 15 min and the fermentation broth was collected for qPCR analysis of VFs and MGEs. The fermentation medium and reducing solution were prepared according to the established method (18). Based on the content of SCFAs in the feces of the high-fiber diet group, we determined the final concentrations of AA, PA, and BA added in the *in vitro* fermentation, which were 70 μmol/mL, 30 μmol/mL, and 20 μmol/mL, respectively.

### Statistics

Data preprocessing was performed using Excel 2019 (Microsoft, United States), and data statistics were performed using SPSS 22.0 (IBM Corp., United States). Graphical display of results was performed using GraphPad Prism 10. RPKM value was used for heat map data, *Z*-score was used for data standardization, and average clustering was used for cluster analysis. Correlation analysis used Spearman. The “protest” function in the vegan package was used to analyze the Procrustes correlation between the bacteriome and the VFs. Principal coordinate analysis (PCoA) and non-metric multidimensional scaling (NMDS) analyses were performed using the pure vegetation package in R software, using VFs and normalized abundance values of bacterial communities with a Bray–Curtis distance. Distribution of VFs in bacteria of different taxonomic levels was plotted as a Sankey diagram using the networkD3 package^1^ in R (v3.6.2). Mantel test correlation heatmaps were plotted using the ggplot2 package (3.3.3) in R (v4.1.3) and the two matrix correlations were tested using the linkET package (0.0.7.4). The mulberry graph is drawn using the plotly package in the R software. Circos analysis was produced and analyzed using the tools of the Megi Bio cloud platform.

Reads per kilobase million (RPKM):


$${\mathrm{RPKM}}_{i}=\frac{R_{i}*10^{6}}{L_{i}*\sum_{1}^{n}\left(R_{j}\right)}$$

$R_{i}$ represents the abundance value of Genei in a sample, that is, the number of reads aligned to Genei in the sample; $L_{i}$ represents the nucleotide length of Genei; $\overset{n}{\underset{1}{\sum }}\left(R_{j}\right)$ represents the sum of the reads corresponding to all genes in the sample.

Experimental design using a 3 × 2 design, two-way ANOVA was used to analyze two main effects and interaction effects. Results were expressed as mean ± standard error, and *p < 0.05* indicated a significant difference.

## Result

### Fiber diet and species-mediated changes in the spectrum of VFs

**Figure 1:**
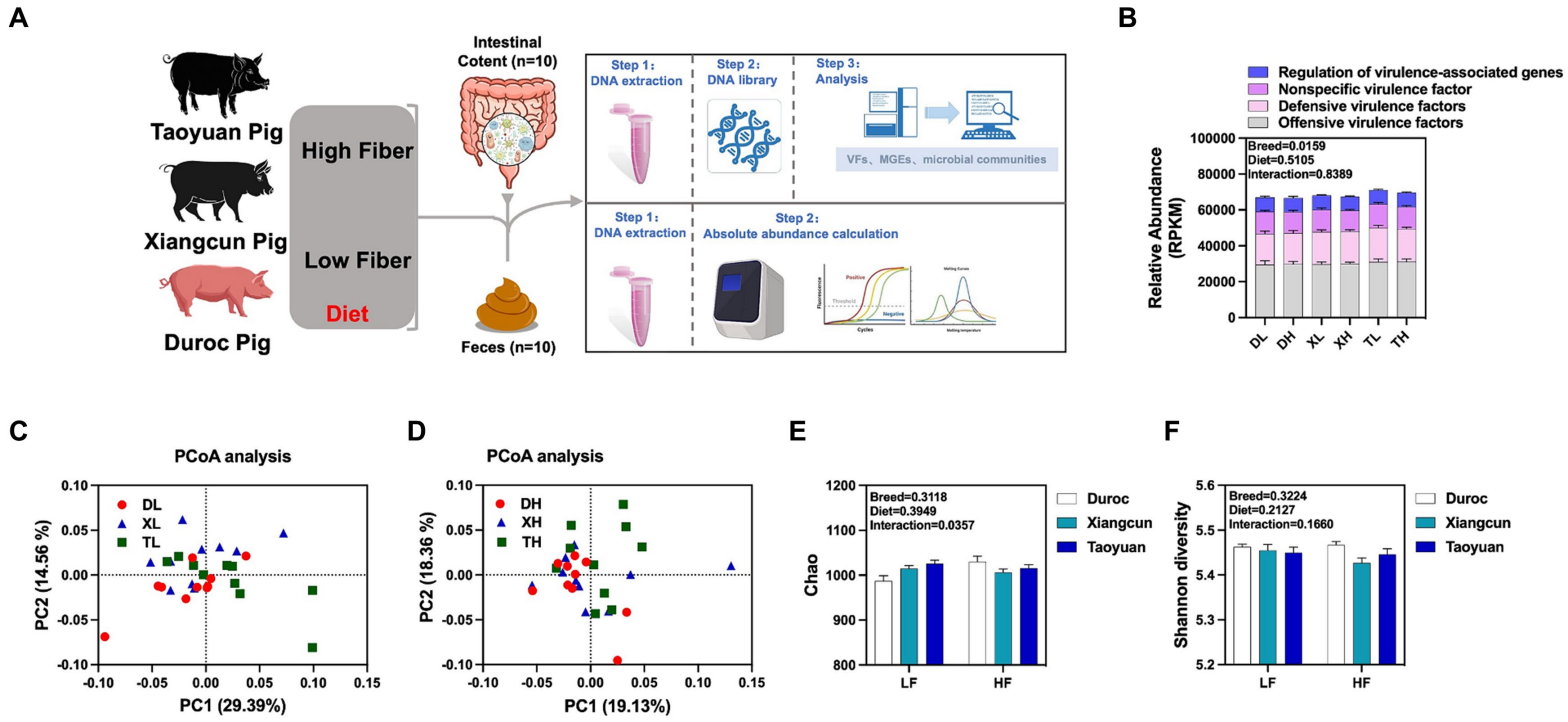
Effects of different fiber levels on the abundance and diversity of VFs in the colonic contents of different breeds of pigs. Experimental flow chart **(A)**. Total abundance of VFs in colonic contents **(B)**. PCoA analysis **(C,D)** and alpha diversity analysis **(E,F)** of VFs in the colonic contents of different pig breeds at high and low fiber levels. ^*^p < 0.05.

**Figure 2:**
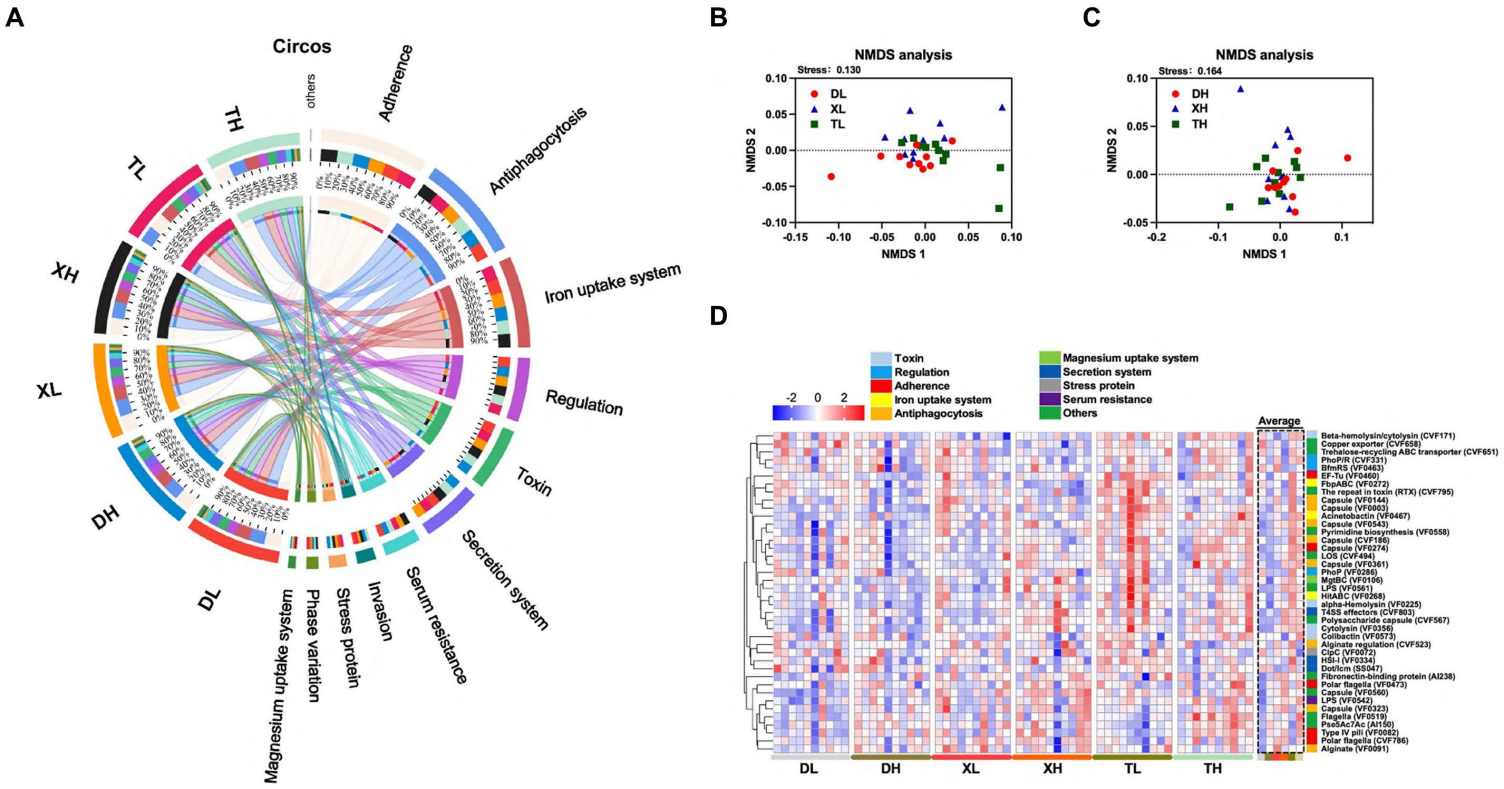
Effect of different fiber levels on the composition of VFs in the colonic contents of different breeds of pigs. Circos analysis of VFs in colonic contents **(A)**. NMDS analysis of VFs in the colonic contents of different breeds of pigs at high and low fiber levels **(B,C)**. Heatmap of VFs enrichment analysis of the top 40 genes with relative average abundance in different samples and groups **(D)**. Different colors on the left side of the heat map indicate different categories of VFs.

To research the effects of fiber diet and genetics on the diversity, abundance and distribution characteristics of VFs in the colonic contents of different breeds of pigs, we performed metagenomic analyses of the colonic contents of Duroc, Taoyuan and Xiangcun pigs on a high and low fiber diet (Figure 1A). In the colonic contents, 1,236 VFs were identified (Supplementary Table S1). The mechanistic classification revealed that both Offensive and Defensive virulence factors predominated, constituting over 70% of VFs. There was no significant disparity in the overall VF abundance across varying fiber levels. Yet, distinct differences in VF abundance were evident between breeds, with Taoyuan and Xiangcun pigs showcasing heightened VF levels (Figure 1B). PCoA analysis disclosed distinctive clustering for Duroc and Taoyuan pigs across fiber levels, while Xiangcun pigs resembled both breeds to some extent (Figures 1C,D). Through α diversity analysis, we discerned no noteworthy differences in VF abundance or diversity concerning fiber levels or breeds (Figures 1E,F). At level 2, VFs were segregated into 16 categories based on biological characteristics. The most abundant factors included adherence, antiphagocytosis, iron uptake system, and others (Figure 2A; Supplementary Figure S1). The high-fiber diet notably diminished the abundance of the iron uptake system and Ig protease (Supplementary Figure S1; Supplementary Table S2). Between breeds, Taoyuan pigs exhibited significantly higher abundance levels of the six virulence factor categories compared to Duroc pigs (Supplementary Figure S1; Supplementary Table S2). To overcome the shortcomings of linear models and better reflect the non-linear structure, we evaluated the accuracy of the model with NMDS stress values. We confirmed that the stress values less than 0.2, which ensured the reliability of the model. The NMDS analysis showed that in the low-fiber group, Duroc and Taoyuan pigs formed clearly differentiated clusters, whereas Xiangcun pigs showed some degree of similarity with both Duroc and Taoyuan pigs. In the high-fiber group, clusters of different breeds were more tightly clustered together (Figures 2B,C).

Expanding our study into the influences of breed and fiber on pig intestinal VFs, we examined the abundances of the top 40 VFs. The analysis revealed inter-breed differences in 18 of the top 40 VFs, with Taoyuan pigs exhibiting higher abundances compared to Duroc pigs (Figure 2D; Supplementary Table S3). These differences including *LOS (CVF494), Capsule (VF0543), Beta-hemolysin/cytolysin (CVF171), LPS (VF0542), HitABC (VF0268), FbpABC (VF0272) Capsule (VF0274), Polysaccharide capsule (CVF567), Fibronectin-binding protein (AI238), Cytolysin (VF0356), Pyrimidine biosynthesis (VF0558), alpha-Hemolysin (VF0225), T4SS effectors (CVF803), Capsule (VF0003), The repeat in toxin (RTX) (CVF795), EF-Tu (VF0460), Acinetobactin (VF0467)* and *Capsule (VF0560)*. In an analysis of different fiber levels, a high-fiber diet significantly reduced 7 of the top 40 enriched VFs *(HitABC (VF0268), FbpABC (VF0272), alginate conditioning (CVF523), capsule (VF0144), encapsulant (VF0003), RTX (CVF795),* and *copper exporter (CVF658))* (Figure 2D; Supplementary Table S3). The results showed that the abundance of VFs was higher in the colonic contents of local pigs under the same scale feeding pattern, while the high-fiber diet reduced the abundance of some VFs.

### Relationship between bacterial classification kinetics and virulence factors

**Figure 3:**
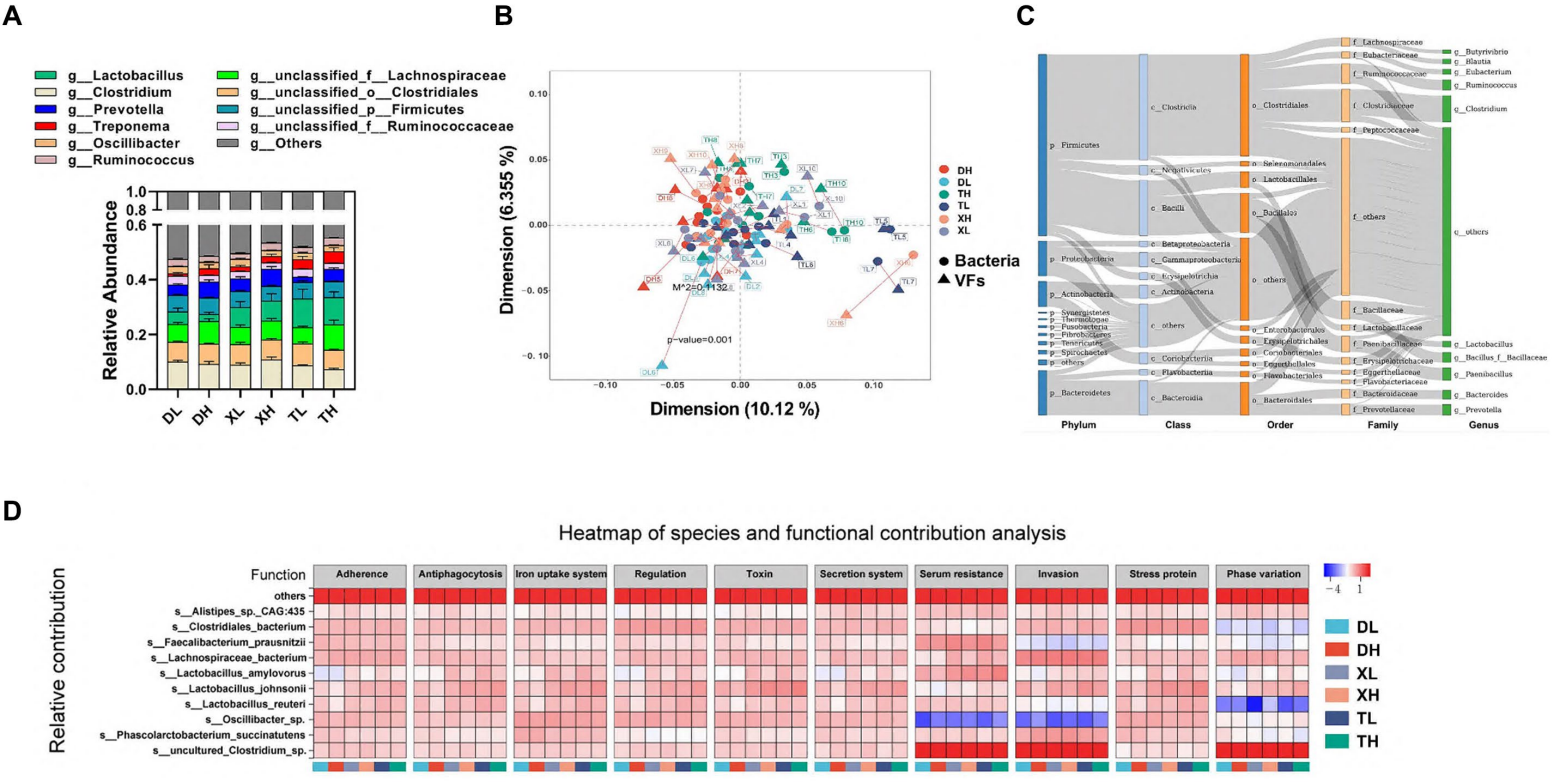
Correlation of VFs with gut microbes. Genus-level microbial composition of pig colon contents from different groups **(A)**. Procrustes analyzed the correlation between intestinal bacterial species and abundance of VFs **(B)**. Dots indicate the ranked position of bacterial species abundance in each sample, and triangles indicate the ranked position of VFs abundance. The length of the line between the dots and triangles shows the Procrustes residuals. **(C)** Distribution of VFs across different classes of bacteria (unclassified microorganisms were removed from the analysis). The colors of the rectangles represent the different classification levels. The length of the rectangle represents the number of VFs. Top 10 species-level microorganisms contributing to VFs abundance **(D)**.

Intestinal VFs predominantly originate from gut microbiota. These were analyzed through a comparative assessment of metagenomic microbial data against the VFDB database. The top 10 genus-level microorganisms present in the porcine colon, constituting over 40% of the population, included *Lactobacillus, Clostridium, Prevotella, Treponema, Oscillibacter, Ruminococcus, unclassified_f_Lachnospiraceae, unclassified_o_Clostridiales, unclassified_p_Firmicutes,* and *unclassified_f_Ruminococcaceae* (Figure 3A). Alpha diversity analysis indicated that a high-fiber diet substantially enhanced microbial diversity, with no marked disparities across species. A Procrustes analysis revealed a significant correlation between VFs and bacterial taxa, showing that VFs composition aligned closely with bacterial composition (*m*^2^ = 0.1132, *p* = 0.001) (Figure 3B). VFs were widely distributed among a variety of bacteria, mainly from the phylum *Firmicutes* (*Clostridium* and *Bacillus*), the phylum *Bacteroidetes* (mainly *Bacteroidia* and *Flavobacteriia*), the phylum *Proteobacteria* (mainly *Gammaproteobacteria* and *Betaproteobacteria*), and the phylum *Actinobacteria* (mainly *Actinobacteria* and *Coriobacteriia*) (Figure 3C). Correlation analysis between species abundance and functional abundance was performed based on the relative species and functional abundance of the samples. We identified species-level microorganisms that were major contributors in the abundance of VFs, which were mainly *s_Lactobacillus_johnsonii*, *s_Clostridiales_bacterium,* s*_Oscillibacter_sp., s_Lachnospiraceae_bacterium, s_Lactobacillus_reuteri, s_uncultured_Clostridium_sp., s_Phascolarctobacterium_succinatutens, s_Lactobacillus_amylovorus, s_Faecalibacterium_prausnitzii*, and *s_Alistipes_sp._CAG:435* (Figure 3D).

**Figure 4:**
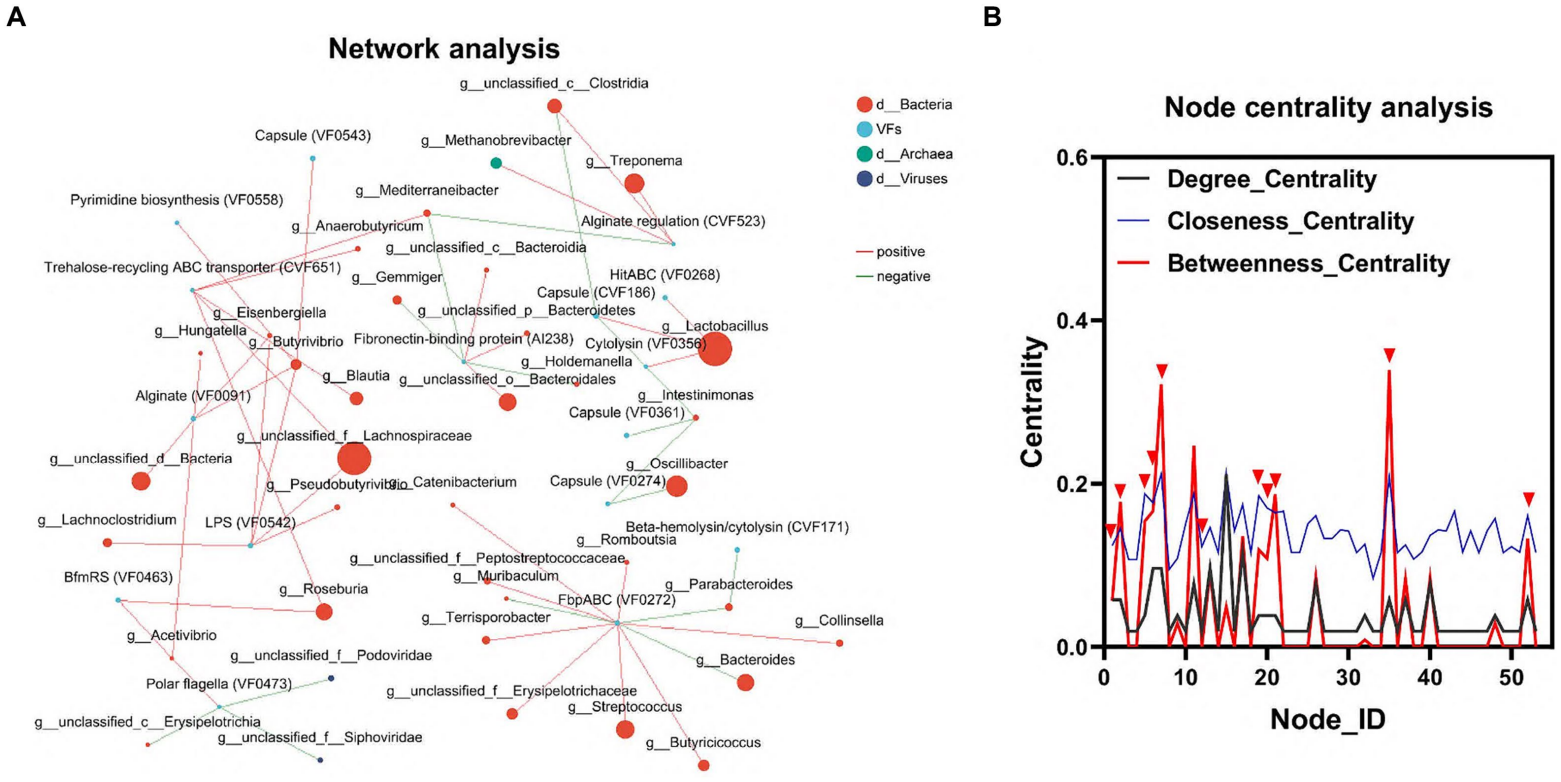
Interaction of colonic microbiota and VFs. **(A)** Network diagram of the interaction between the dominant microbiota and VFs. **(B)** Critical strain nodes of the core genus were analyzed. Black curves indicate degree centrality. Blue curves indicate proximity centrality and red curves indicate mediated centrality. Nodes where all three curves peak simultaneously may be genera that play an important role in the interaction network.

To elucidate the relationship between gut flora and VFs, we conducted an interaction network analysis using Network software. This analysis aimed to assess the interactions and correlations between the top 100 genera of microorganisms and VFs within the same sample. In the node centrality plot, the numbers on the *x*-axis represent different nodes. Each node corresponds to the interaction of a single genus with other genera. When a peak appears simultaneously on all three curves between *Degree_Centrality, Closeness_Centrality* and *Betweenness_Centrality,* we consider that the genus corresponding to that node may be important for the whole network. Based on this principle, we identified 6 genera, including *g_Lactobacillus, g_unclassified_f_Lachnospiraceae, g_Roseburia, g_unclassified_c_Clostridia, g_ Mediterraneibacter,* and *g_Acetivibrio*, as well as three VFs, including *LPS (VF0542)*, T*rehalose-recycling ABC transporter (CVF651),* and *BfmRS (VF0463).* This result further confirmed the important influence of gut flora on the distribution of VFs (Figures 4A,B; Supplementary Table S4).

### Integrated analysis of microorganisms, SCFAs and MGEs with virulence factors

**Figure 5:**
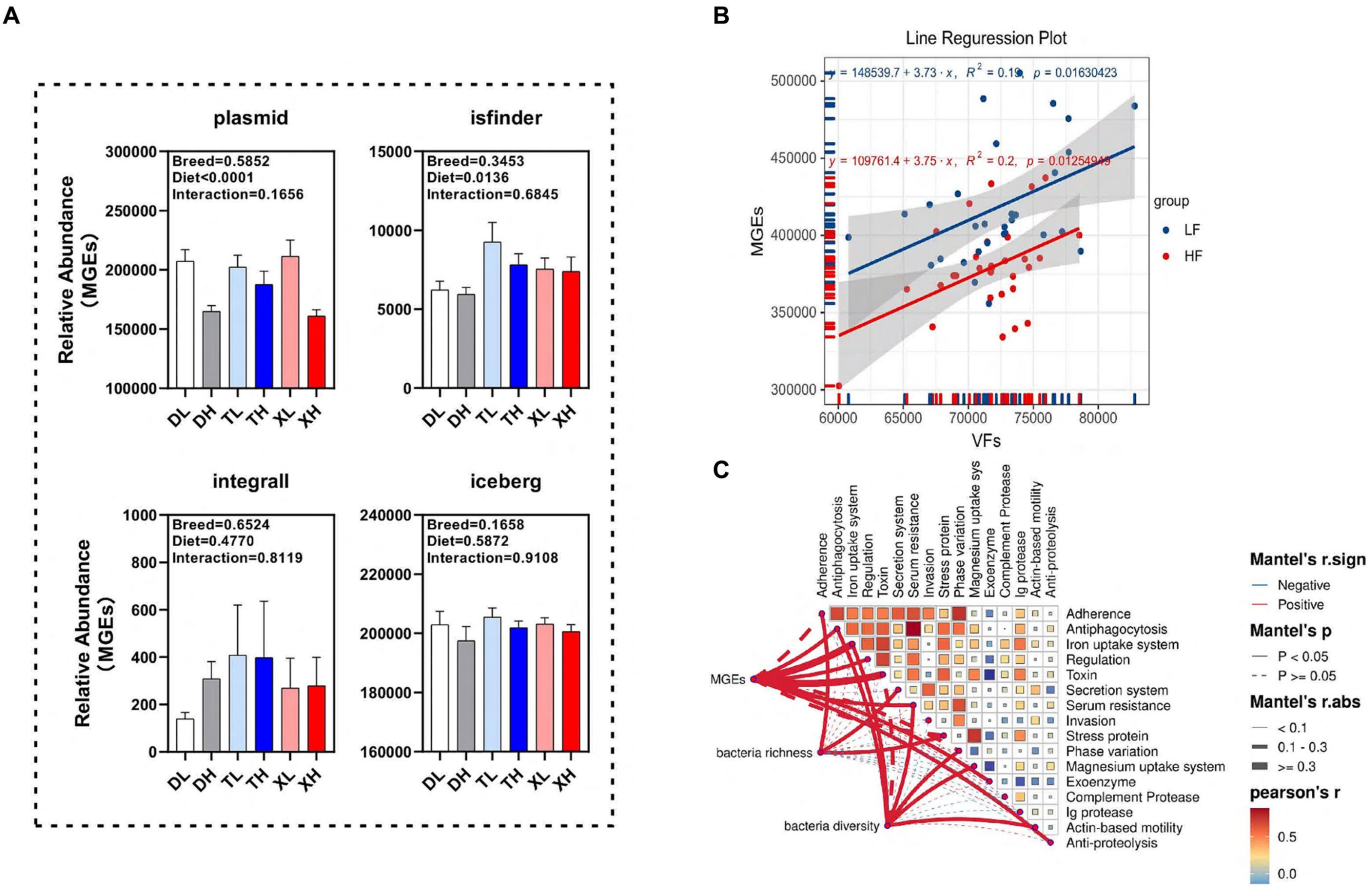
Potential drivers mediating changes in VFs. **(A)** Differences in different classes of MGEs (plasmid, isfinder, integrall, and iceberg) in the colonic contents of different groups of pigs. Linear regression analysis of the relative abundance of VFs and MGEs in colonic contents at different fiber levels **(B)**. Mantle analysis of MGES, microbial abundance and microbial diversity and different categories of VFs **(C)**.

We examined the disparities in the abundance of functional classes of MGEs in the colonic contents of different porcine breeds subjected to varying fiber intake levels. Our findings revealed that a high-fiber diet markedly diminished the relative abundance of plasmids (*p* < 0.05) without affecting isfinder, integrall, or iceberg. Interestingly, the MGEs categories within the porcine colonic contents showed no significant variations across breeds (Figure 5A). Linear correlation analysis indicated a notable positive association (*p* < 0.05) between the abundances of MGEs and VFs (Figure 5B). To determine the correlation of microbial diversity and MGEs with VFs, we assessed the relationship between microbial diversity and abundance of MGEs and different classifications of VFs. The results of the Mantel assay showed that MGEs were associated with *Antiphagocytosis*, Iron uptake system, Regulation, Toxin, Serum resistance, Exoenzyme and Anti-proteolysis, Bacteria richness was significantly positively correlated with Adherence, Secretion system and Stress protein, bacteria diversity was significantly positively correlated with *Antiphagocytosis*, Iron uptake system, Serum resistance, Phase variation, Magnesium uptake system and Actin-based motility (Figure 5C).

### Effects of breed and fiber diets on the distribution of VFs in swine manure and their potential drivers

**Figure 6:**
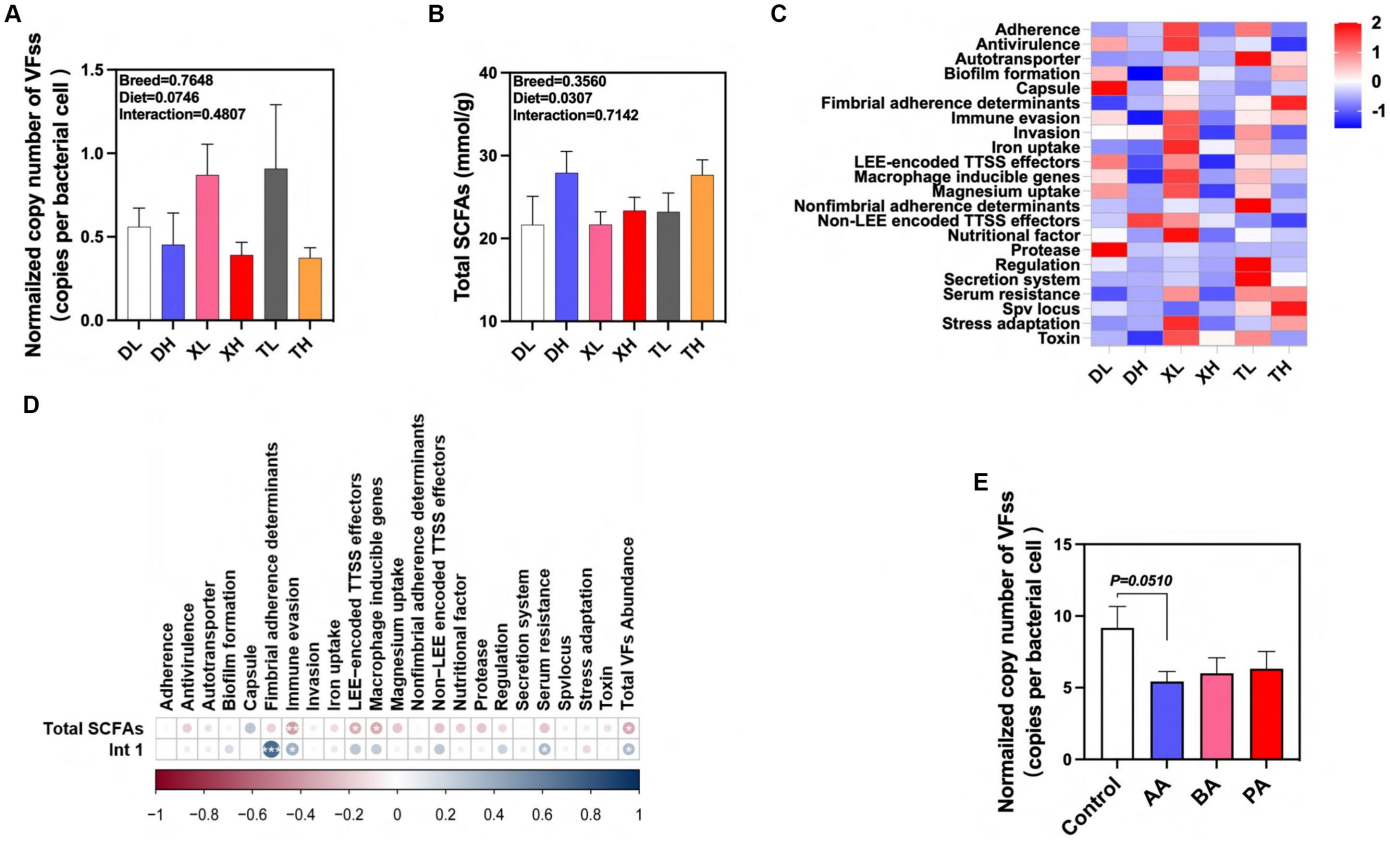
Effects of different fiber levels and species on excretion of VFs in feces and their drivers. **(A)** Normalized abundance of VFs in fecal samples. Total SCFA in fecal samples **(B)**. **(C)** Heatmap of enrichment analysis of different classes of VFs in feces from different groups of pigs. **(D)** Correlation of VFs with intI1 gene and total SCFAs in fecal samples. **(E)** Effect of AA, BA and PA on the standardized abundance of VFs under *in vitro* fermentation mode. aA, acetic acid; PA, propionic acid; BA, butyric acid; VA, valeric acid; IPA, isopropionic acid; IBA, isobutyric acid; IVA, isovaleric acid. ^*^p < 0.05, ^**^p < 0.01, and ^***^p < 0.001. Color change indicates *R* value.

We detected 46 subtypes of VFs in the feces of different breeds of pigs by absolute quantification. These VFs were mainly classified into 22 categories by function, including Adherence, Antivirulence, Autotransporter, Biofilm formation, Capsule, Fimbrial adherence determinants, Immune evasion, Invasion, Iron uptake, LEE-encoded TTSS effectors, Macrophage inducible genes, Magnesium uptake, Nonfimbrial adherence determinants, Non-LEE encoded TTSS effectors, Nutritional factor, Protease, Regulation, Secretion system, Serum resistance, Spv locus, Stress adaptation and Toxin (Figure 6A). The results showed that high fiber diets significantly reduced the normalized abundance of total VFs in swine feces, and there was no significant difference in the abundance of VFs between breeds (Figures 6A,C). Previous laboratory studies have shown that high-fiber diets resulted in a significant reduction in the normalized abundance of the integrase gene *intI1* in feces (19). After examining the amount of SCFAs in feces we found that the high fiber diet significantly elevated the amount of total SCFAs in swine feces, but there were no differences between breeds (Figure 6B). We used correlation analysis to further research these relationships. We found significant positive correlations between the normalized abundance of *intI1* and the categories of *Fimbrial* adherence determinants, Immune evasion, Serum resistance, and Total VFs Abundance, whereas the fecal concentration of Total SCFAs was significantly correlated with the concentrations of Immune evasion, LEE-encoded TTSS effectors, Macrophage inducible genes and Total VFs Abundance categories showed significant negative correlations with each other (Figure 6D). In contrast, high-fiber dietary interventions may effectively modulate fecal excretion of VFs through the intermediate effects of their metabolic byproducts, SCFAs and MGEs. To confirm the association between SCFAs and VFs, we evaluated the effects of BA, AA, and PA on VFs carried by microorganisms after fecal flora fermentation using an *in vitro* fermentation model. The results showed that AA tended to reduce the normalized abundance of VFs in swine fecal fermentation broth (*p* = 0.051) (Figure 6E). In conclusion, our results suggest that fiber diets can influence the excretion of VFs in different pig breeds through the intermediate action of their metabolite AA. In addition, this effect can inhibit the spread of VFs by reducing MGEs in feces.

## Discussion

VFs harbored by pathogenic bacteria pose significant health challenges, complicating disease management. The VFs present in gut and fecal flora are significant environmental sources (20). Grasping the distribution of these VFs aids in managing potential human pathogens, with their abundance and spread influenced by genetic, environmental, and dietary pressures (21).

Under intensive feeding conditions, Taoyuan pigs exhibit a higher abundance of virulence factors (VFs) in their intestinal contents, and a high-fiber diet has been observed to reduce the relative abundance of certain highly abundant VFs. This phenomenon may be attributed to the presence of diverse pathogenic microorganisms in the intestinal tracts of local pig breeds, influenced by their natural characteristics and genetic backgrounds. Moreover, the decline in immune performance during large-scale breeding could lead to the heightened expression of virulence factors (22). Some studies have shown that wild giant pandas have more pathogenic bacteria carrying virulence factors compared to captive giant pandas (23). Intriguingly, our findings reveal that the predominant function of the highly expressed VFs in the intestines of Taoyuan pigs, in contrast to Duroc pigs, is to facilitate easier colonization of pathogenic bacteria in the host intestinal tract, evade host immunity, and enhance iron uptake. This underscores the higher environmental resistance and adaptability of pathogenic microorganisms in the intestinal tracts of endemic pigs such as Taoyuan pigs. Studies have shown that changes in metabolism significantly affect the distribution and abundance of pathogenic bacteria in animals (24). Dietary fiber, known to play a crucial role in energy metabolism, lipid metabolism, and glucose metabolism in animals, has been associated with altering disease outcomes (25). It has been demonstrated that some plant extracts, such as populin and quercetin, can improve disease outcomes in *S. aureus* patients by reducing *S. aureus* virulence (26). The results of the present study showed that a high-fiber dietary regimen could significantly reduce the relative abundance of some VFs. And the main functions of the reduced VFs were pathogenic bacteria colonization in the host intestine, evasion of host immunity and iron absorption-related VFs.

The colon microbiota carries many VFs. We should note that gut microbes are the main hosts of VFs, and a higher diversity of gut microbes implies multiple genetic mechanisms and more types of VFs that are the source of many unknown virulence factors (27). This may explain the high levels of VFs in the colonic contents of Taoyuan and Xiangcun pigs. A limited number of microbial species were identified as contributing many VFs. *s__Phascolarctobacterium_succinatutens* was found to contribute an abundance of VFs. *s__Phascolarctobacterium_succinatutens* was found to be the culturable species-level microorganism carrying the most VFs in the pig colon. Several previous studies have reported that *s__ Phascolarctobacterium_succinatutens* was enriched with in the intestines of patients with liver cirrhosis and colon cancer, among others (28, 29). This suggests that *s__Phascolarctobacterium_succinatutens* may be a potential causative agent for certain diseases. Additionally, it is noteworthy that various microorganisms belonging to the genus *Lactobacillus* also harbor significant quantities of VFs. Some studies have even confirmed the presence of numerous resistance genes in the genome of the genus *Lactobacillus* (30). This underscores the importance of considering potential safety risks associated with the use of Lactobacillus in food fermentation processes.

Microorganisms, MGEs and SCFAs are important drivers of changes in VFs in the gut. VFs carried by MGEs undergo constant horizontal shifts and changes in abundance through human induction and environmental changes (31). We observed a linear and positive correlation between the relative abundance of MGEs and VFs. As a key reason for their survival and spread, MGEs have been determined to be horizontally transmitted to a wide range of bacteria (both pathogenic and human commensal) (32). Numerous VFs are harbored in the intestinal tract of pigs, with MGEs serving as potential vectors for horizontal gene transfer. Consequently, these VFs are likely to be transmitted to humans through the food chain or environmental routes such as rivers and soils, posing potential health risks. In contrast, our study highlights the potential of short-chain fatty acids, essential metabolites derived from high-fiber diets, as a means of mitigating the carriage of VFs in livestock and poultry.

Dietary fiber reduces the abundance of VFs carried by microorganisms in feces through its metabolite SCFAs. Animal feces are produced because of the digestive and metabolic processes of food through the gastrointestinal tract, and therefore alterations in metabolic processes in the gut may influence fecal properties (33), which may include the abundance of VFs. Thus, the observed effect of a fiber-rich diet on the abundance of VFs in feces may be influenced by the role of fiber-derived metabolites in the gut. Consistent with previous studies, our findings showed that a fiber diet significantly increased the fecal content of total SCFAs (34). In addition, significant negative correlations were found between total SCFAs and the abundance of functional classes of virulence factors, suggesting that a high-fiber diet may influence the excretion of VFs in feces through their metabolites SCFAs. Therefore, we further demonstrated that AA tended to reduce the abundance of VFs by validation of an *in vitro* fermentation model. These results suggest to us that AA may have the effect of reducing VFs by inhibiting the proliferation of VFs host microorganisms (e.g., pathogenic microorganisms in feces). In addition, through the successful application in fermentation modeling, we also recognized that AA could realize its potential to promote environmental safety by being added to agricultural production processes such as composting.

## Conclusion

This research offers an exhaustive analysis of the influence of genetics and dietary fiber on the distribution, composition, and drivers of virulence factors (VFs) in pig colonic contents and feces. It was observed that the indigenous Chinese breed, Taoyuan pig, exhibited a higher relative abundance of VFs than the commercial Duroc pig in large-scale farming. Notably, the VFs profiles differed significantly between the two breeds. The intracolonic VFs of the hybrid “Xiangcun pig” melded characteristics from both Taoyuan and Duroc strains. While these insights are invaluable for gauging VFs shifts during breeding, a broader and more diverse sample is essential for thoroughly understanding VFs transmission and alterations. High-fiber diets marginally reduced VFs abundance in colonic contents and, to a lesser extent, in feces. The primary contributors to these changes were microbes, MGEs (predominantly plasmids), and SCFAs (chiefly AA). Given the prevalent use of fiber diets and the metabolite AA in production, this establishes a solid foundation for VFs risk mitigation in swine farming environments. The results of this study can be used as a reference for optimizing feed strategies and reducing the risk of foodstuffs carrying VFs.

## Data availability statement

The original contributions presented in the study are publicly available. This data can be found at: https://www.ncbi.nlm.nih.gov, BioProject: PRJNA849732.

## Ethics statement

The animal study was approved by Institutional Animal Care and Use Committee of Sichuan Agricultural University, Sichuan, China (No. 20181105). The study was conducted in accordance with the local legislation and institutional requirements.

## Author contributions

TW: Conceptualization, Data curation, Formal analysis, Investigation, Methodology, Validation, Visualization, Writing – original draft, Writing – review & editing. YL: Conceptualization, Data curation, Methodology, Visualization, Writing – original draft, Writing – review & editing. XK: Conceptualization, Data curation, Formal analysis, Methodology, Writing – review & editing. BY: Conceptualization, Investigation, Methodology, Project administration, Writing – review & editing. PZ: Conceptualization, Investigation, Methodology, Project administration, Writing – review & editing. ZH: Conceptualization, Investigation, Methodology, Project administration, Writing – review & editing. XM: Conceptualization, Investigation, Methodology, Project administration, Writing – review & editing. JY: Conceptualization, Investigation, Methodology, Project administration, Writing – review & editing. JL: Conceptualization, Investigation, Methodology, Project administration, Writing – review & editing. HY: Conceptualization, Investigation, Methodology, Project administration, Writing – review & editing. JH: Conceptualization, Methodology, Resources, Supervision, Writing – original draft, Writing – review & editing.

## Footnotes

^1^ https://cran.r-project.org/web/packages/networkD3
